# Exposure to previous cART is associated with significant liver fibrosis and cirrhosis in human immunodeficiency virus-infected patients

**DOI:** 10.1371/journal.pone.0191118

**Published:** 2018-01-18

**Authors:** Evrim Anadol, Kristina Lust, Christoph Boesecke, Carolynne Schwarze-Zander, Raphael Mohr, Jan-Christian Wasmuth, Jürgen Kurt Rockstroh, Jonel Trebicka

**Affiliations:** 1 Department of Internal Medicine I, University Hospital Bonn, Bonn, Germany; 2 German Centre for Infection Research (DZIF), partner site Bonn-Cologne, Bonn, Germany; 3 Faculty of Health Sciences, University of Southern Denmark, Odense, Denmark; 4 European Foundation for the Study of Chronic Liver Failure—EF Clif, Barcelona, Spain; 5 Institute for Bioengineering of Catalonia, Barcelona, Spain; Medizinische Fakultat der RWTH Aachen, GERMANY

## Abstract

**Introduction:**

Combined antiretroviral therapy (cART) has improved survival in HIV-patients. While the first antiretrovirals, which became available in particular D-drugs (especially didanosine and stavudine) and unboosted protease inhibitors, may impair liver function, the modern cART seems to decrease liver fibrosis. This study assessed the influence of exposure to previous antiretrovirals on liver fibrosis in HIV-infected patients.

**Methods:**

This observational cross-sectional single-center study recruited 333 HIV patients and assessed liver fibrosis using transient elastography (TE).

**Results:**

83% were male with a median age of 45, while 131 were co-infected with viral hepatitis. Overall, 18% had significant fibrosis and 7.5% had cirrhosis. 11% of HIV mono-infected patients had significant fibrosis and 2% had cirrhosis. HCV infection (OR:5.3), history of exposure to didanosine (OR:2.7) and HIV load below 40copies/mL (OR:0.5) were independently associated with significant fibrosis, while HCV (OR:5.8), exposure to didanosine (OR:2.9) and azidothymidine (OR:2.8) were independently associated with cirrhosis. Interestingly, in HIV mono-infected patients, a HIV-load below 40copies/mL (OR:0.4) was independently associated with significant fibrosis, and didanosine (OR:20.8) with cirrhosis.

**Conclusion:**

In conclusion, history of exposure to didanosine and azidothymidine continues to have an impact on the presence of liver cirrhosis in HIV patients. However, HCV co-infection and ongoing HIV-replication have the strongest effect on development of significant fibrosis in these patients.

## Introduction

Progressive liver injury is a growing concern in HIV-infected patients not only due to co-infection with hepatitis B (HBV) or C (HCV) viruses and alcohol overuse, but also as a result of long-term exposure to antiretroviral drugs [1, 2]. While modern combined antiretroviral therapy (cART) reportedly has mostly beneficial effects on liver fibrosis [3], older antiretrovirals have been shown to cause severe complications of liver fibrosis and portal hypertension (e.g. bleeding associated to didanosine (ddI) exposure) [4–7]. In most HIV infected patients, effective combined antiretroviral treatment leads to a markedly increased life expectancy, while in HBV or HCV co-infected patients, liver-associated mortality has become a major cause of death [8, 9]. The accelerated progression of liver disease in HIV/HCV co-infected patients is prevented by cART [10, 11], suggesting that fibrogenesis may be induced by HIV itself, probably through directly affecting hepatic stellate cells [3, 12–14].

The prevalence of significant liver fibrosis among HIV mono-infected patients has been reported to be about 11–16% when assessed by noninvasive liver stiffness measurement [15, 16]. Moreover, chronic and intermittent elevations of alanine aminotransferase (ALT) have been described in up to 19% of HIV mono-infected patients during ART, possibly mirroring drug induced liver injury (DILI) [16–18]. Accordingly, long-term exposure to cART may contribute to liver injury and drive fibrogenesis. Also, the influence of previous exposure to older drugs on the presence of liver fibrosis or cirrhosis progression has not been completely clarified.

Therefore, the aim of this study was to assess the influence of a history of previous antiretrovirals on the presence of significant fibrosis and cirrhosis in HIV-infected patients.

## Material and methods

### Study population and design

In this cross-sectional study, 333 HIV-infected patients of the HIV Outpatient Clinic at the Bonn University Hospital were enrolled who regularly attended the clinic for follow up between August 2009 and December 2011. Written informed consent was obtained prior to study onset. Our local ethics committee (full name: **Ethikkommission an der Medizinischen Fakultät der Rheinischen Friedrich-Wilhelms-Universität Bonn**) in accordance with the Declaration of Helsinki had approved the study protocol (No. 069/10). In all patients, liver fibrosis was assessed non-invasively by transient elastography (TE) using FibroScan®. Demographic and clinical data, such as Centers for Disease Control and Prevention (CDC) category illness and risk factors for liver fibrosis were recorded in all patients. Alcohol overuse was defined as an average daily consumption > 30g (more than two standard drinks) either at enrollment or in the past six months.

Details of cART (combined antiretroviral therapy) including total duration of treatment and current regimens based on liver stiffness measurement (LSM) were recorded.

The blood samples were collected together with the periodically assessed routine laboratory parameters, including CD4+ T-cell count, HIV-RNA viral loads (VL) and liver function tests. Serum HCV-RNA, HCV antibody status and serum hepatitis B surface antigen (HBsAg) detected HCV or HBV infection, which were investigated at the time of inclusion in the cohort as well as whenever indicated after enrollment. Ultrasonography evaluation was performed as part of routine evaluation at enrollment. A gastroscopy was performed upon clinical or ultrasonography signs of portal hypertension. Abdominal paracentesis was performed in patients with ascites prior to TE. All methods were performed in accordance with the relevant guidelines and regulations.

### Transient elastography (TE) and ultrasonography examination

Transient elastography was performed with FibroScan^**®**^ (FibroScan^**®**^ 502; Echosens, Paris, France) by an experienced physician according to the instructions provided by the manufacturer. An ultrasound transducer probe tip is placed on the skin of the patients between the rib bones over the right lobe of the liver, which transmits an elastic shear wave that propagates through the liver tissue. The velocity of this wave, which is directly related to tissue stiffness, is measured by ultrasound [19].

After obtaining at least ten measurements, the median of which, expressed in kilopascal (kPa) units was assumed as representative of overall TE. Transient elastography findings with a success rate of at least 60% (number of validated measurements divided by the total number of measurements) and an interquartile range (IQR) to median value ratio of less than 30% were regarded as valid [20]. A cut-off of ≥ 7.1 kPa was defined as significant fibrosis and ≥ 12.5 kPa as cirrhosis according to previous recommendations [18, 19].

### Non-invasive indeces of liver fibrosis

FIB-4 index and APRI score were calculated as previously described [21]. The FIB-4 index is based on AST, ALT, platelet count, and age and calculated as: FIB4 = (age[year] X AST[IU/L]) / (platelet count[10^9^/L] X SQR(ALT[IU/L])). The APRI score was calculated as: APRI = ((AST / ULN) /platelet count [10^9^/L]) X 100. Relevant liver fibrosis was defined as FIB-4-index > 1.45 and APRI > 1.

### Statistical analysis

Statistical analysis was performed using SPSS Statistics, version 20 for Windows (SPCC, Chicago, IL, USA). Continuous variables were expressed as median and interquartile range (IQR). Categorical data were expressed as numbers and percentages. The main characteristics of patients with cirrhosis or fibrosis and of those without were compared using Kruskal-Wallis-Test, Mann-Whitney-U-Test or chi-squared and trend test as appropriate. In addition, univariate and multivariate models were employed to determine which factors were independently associated with significant fibrosiss assessed as transient elastography ≥ 7.1 kPa using binary logistic regression forward step-wise likelihood quotient. Variables with a p-value below 0.05 in the univariate analysis were included in a multivariate regression model. Differences between groups were considered to be significant when p-values were below 0.05.

## Results

### Clinical characteristics of HIV infection in the study population

The clinical characteristics with regard to HIV infection, stratified by the absence of presence of liver fibrosis and cirrhosis, are summarized in Table 1. Briefly, according to transient elastography, 60 (18%) patients had significant fibrosis (7.1–12.4 kPa) and 25 (7.5%) had cirrhosis (≥ 12.5 kPa). The median patient age was 45 years and the vast majority were men. The main transmission risk for more than half of the HIV-infected patients was sexual intercourse with 38% for men who have sex with men (MSM) and 20% for heterosexual transmission. The prevalence of cirrhosis is higher in transfusion-infected patients or former intravenous drug users (Table 1).

**Table 1:** Main HIV-specific characteristics of the study population (n = 333) stratified by grade of fibrosis assessed by transient elastography.

Variable	All patients (n = 333)	TE < 7.1 kPa (n = 248)	7.1≤TE<12.5kPa (n = 60)	TE≥12.5kPa (n = 25)	p-value
**Age [y]**	45 (38–51)	45 (38–50)	45 (41–52)	50 (43–53)	0.067
**Male gender**	275 (83%)	199 (80%)	54 (90%)	22 (88%)	0.09
**BMI [kg/m**^**2**^**]**	24 (22–26)	23 (21–26)	24 (20–26)	25 (23–27)	0.63
**Route of transmission:**	128/67/42/52/44	97/61/23/32/34	27/3/8/15/8	4/3/11/5/2	<0.001
**MSM/HS/TF/IVDA/unknown (%)**	(38/20/12/16/14%)	(39/25/9/13/14%)	(46/5/12/25/12%)	(16/12/44/20/8%)	
**Time since HIV diagnosis [y]**	10 (4–18)	9 (4–15)	11 (6–20)	24 (12–25)	< 0.001
**CDC stage (A / B / C)**	166/91/74	126/68/53	32/ 13/ 14	8/ 10/ 7	n.s.
**(%)**	(50/ 27/ 22%)	(51/ 27/ 21%)	53/ 22/ 23%	32/ 40/ 28%	
**Exposure to cART [n]**	295 (89%)	218 (88%)	52 (87%)	25 (100%)	0.199
**Duration of cART [y]**	5 (2–11)	5 (2–10)	5 (1–13)	10 (4–16)	0.084
**Undetectable HIV load**	262 (79%)	203 (82%)	43 (72%)	16 (64%)	0.016
**CD4 count [cells/μl]**	461 (324–631)	480 (326–630)	455 (361–659)	340 (222–635)	0.32
**PI-based**	183	126	35	22	0.005
**NNRTI-based**	92 (31%)	77 (35%)	14 (27%)	1 (4%)	0.002
**NRTI-based**	19 (6%)	15 (7%)	2 (4%)	2 (8%)	0.830
**AZT**	108 (32.4%)	70 (28.2%)	21 (35%)	17 (68%)	<0.001
**D4T**	86 (25.8%)	59 (23.8%)	15 (25%)	12 (48%)	0.03
**ddI**	41 (12.3%)	21 (8.5%)	10 (16.7%)	10 (40%)	<0.001

Data are shown as median and (interquartile range) or number and (%). Comparisons are performed using Kuskal-Wallis test.

TE = transient elastography; MSM = men who have sex with men; IVDA = intravenous drug abuse; HS = heterosexual; TF = transfusion; kPa = kilopascal; y = years; BMI = Body Mass Index; HIV = human immunodeficiency virus; CDC = Centers for Disease Control and Prevention; cART = combined antiretroviral therapy; AZT = azidothymidine; DDI = didanosine D4T = stavudine.

The median duration of HIV infection was ten years and the median CD4+ T-cell count was 461cells/μl, while the proportion of patients with CD4+ T-cell counts below 200 cell/μl was highest in cirrhotic patients with 20% (5/25) and the median duration of HIV infection was 24 years. In 262 (79%) patients, plasma HIV-RNA load was below the limit of detection and 295 (89%) patients were on cART according to the European Guidelines, while the median time on cART was five years (Table 1).

The proportion of patients with undetectable HIV-load was significantly lower in patients with cirrhosis or significant fibrosis than in patients with neither (p = 0.016). The cART regimen was as follows: PI-based in 60% (177/295), NNRTI-based in 31.2% (92/295) and NRTI-based in 6.4% (19/295). A combination of PI and NNRTI was taken by only 2% (6/295) of patients. Interestingly, a PI-based regimen was present in 88% (22/25) of the cirrhotic patients and in 61.5% (32/52) of patients with significant fibrosis, while NNRTI-based regimen was present in only 26.9% of the patients with significant fibrosis and in 35.3% of the patients without relevant fibrosis (Table 1).

### Clinical characteristics of liver injury in the study population

**Fig 1:**
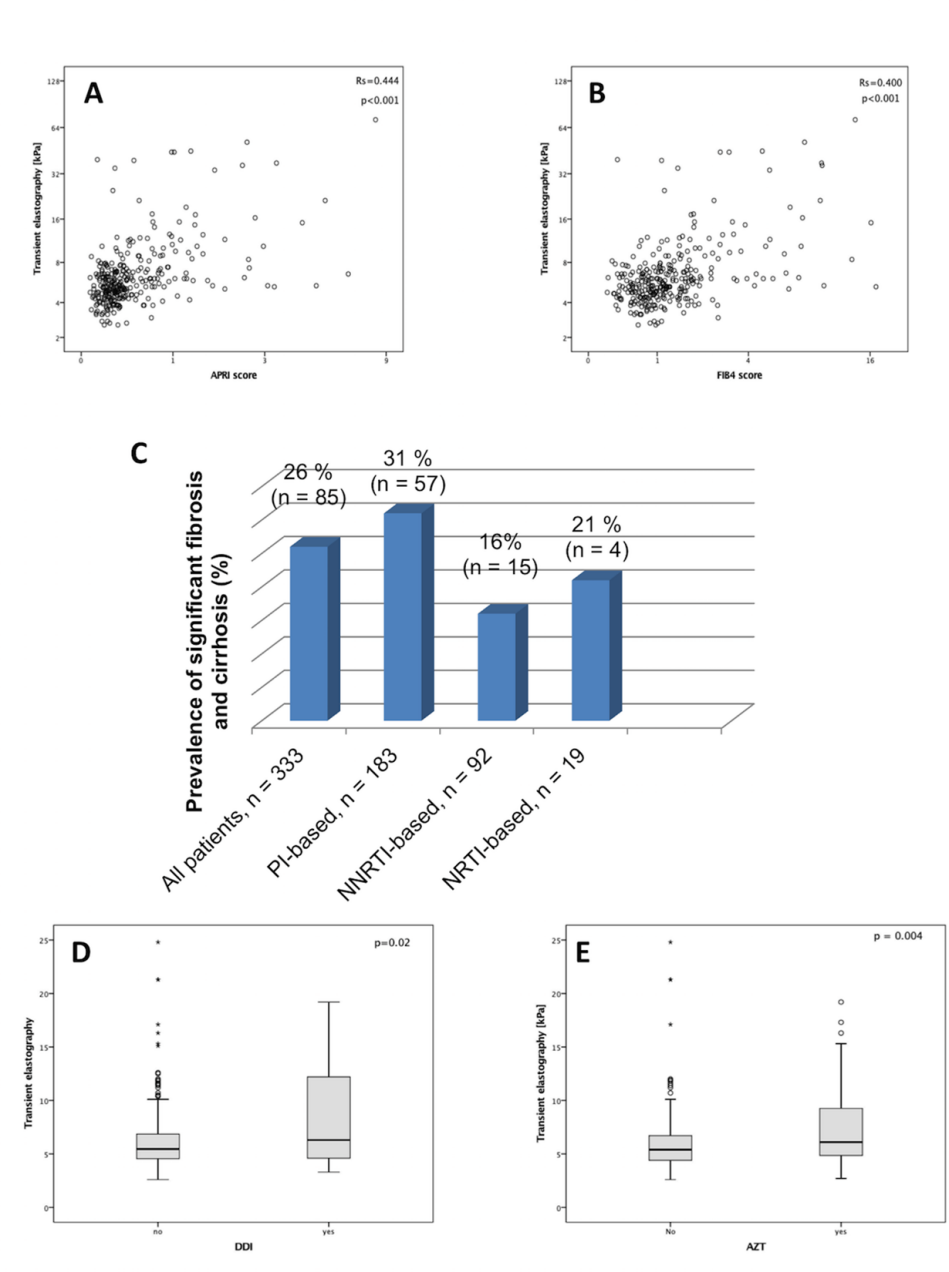
Correlation between TE and APRI score (A) and FIB4 score (B). Panel C depicts the prevalence of fibrosis and cirrhosis stratified by the different cART regimes. The TE levels in patients with and without history of ddI (D) and AZT (E).

The liver-specific characteristics of the cohort are summarized in Table 2. FIB4- and APRI-scores indicated the absence of fibrosis, presence of liver fibrosis and presence of cirrhosis as expected (FIB4: 1 (0.7–1.4) vs. 1.3 (0.9–2) vs. 3.3 (1.9–7.7) and APRI: 0.3 (0.2–0.4) vs. 0.5 (0.3–0.9) vs. 1.2 (0.7–2.4), respectively p<0.001). TE correlated strongly with APRI- and FIB4-score (Fig 1A and 1B), underlining the reliability of the measurements. In the whole cohort, there were 131 (39%) patients with chronic viral hepatitis infection, while HCV-co-infection was present in 112 (34%) patients; HBV-co-infection was found in 19 (6%) patients, five patients had HBV/HCV co-infection and one patient HBV/HCV/hepatitis delta virus (HDV) co-infection. The mean HCV viral load was 1,044,172 copies/ml (range, 3,098–3,796,899). In 67% of the HIV/HBV co-infected (16/24) patients, HBV-RNA was below the limit of detection (34 copies/ml). Of the patients with significant liver fibrosis or cirrhosis, 61.2% were HCV co-infected (Table 2).

**Table 2:** Main liver specific characteristics of the study population (n = 333) stratified by grade of fibrosis assessed by transient elastography.

Variable	All patients (n = 333)	TE < 7.1 kPa (n = 248)	7.1≤TE<12.5kPa (n = 60)	TE≥12.5kPa (n = 25)	p-value
**TE [kPa]**	5.6 (4.6–7.1)	5.1 (4.3–6)	8.7 (7.6–10.1)	21.3 (15.3–39.6)	< 0.001
**HBV co-infection** *	24 (7%)	17 (7%)	4 (7%)	3 (12%)	0.472
**HCV co-infection** *	112 (34%)	58 (23%)	35 (58%)	19 (76%)	< 0.001
**Alcohol consumption > 30g/d**	26 (8%)	16 (62%)	8 (31%)	2 (8%)	0.254
**AST [U/l]**	26 (19–40)	24 (18–33.8)	34 (25–53)	56 (37–67)	< 0.001
**ALT [U/l]**	30 (20–48)	28 (20–43)	44 (22–83)	44 (25–54)	0.007
**y-GT [U/l]**	53 (36–90)	47 (34–79)	71 (45–150)	94 (56–168)	< 0.001
**AP [U/l]**	87 (71–113)	86 (69–111)	90 (69–116)	103 (81–129)	0.032
**Albumin [g/l]**	43.2 (39.7–46)	43.4 (40.5–46.4)	42.6 (39.1–44.6)	39.1 (33.6–42.8)	0.004
**INR**	1.0 (0.9–1.1)	1.0 (0.9–1.0)	1.0 (1.0–1.1)	1.1 (1.0–1.2)	< 0.001
**Platelets [x10**^**3**^**/l]**	206 (164–24)	214 (178–254)	183 (151–225)	111 (54–197)	< 0.001
**Total cholesterol [mg/dl]**	186 (157–215)	188 (164–219)	182 (150–203)	150 (106–185)	0.006
**LDL [mg/dl]**	105 (71–131)	110 (77–134)	105 (75–131)	71(50–185)	0.002
**HDL [mg/dl]**	41 (31–50)	41 (32–51)	41 (31–48)	36 (20–42)	0.057

*five patients had an HBV/HCV co-infection and one of these an HBV/HCV/hepatitis delta virus (HDV) co-infection. Data are shown as median and (interquartile range) or number and (%). Comparisons are performed using Kuskal-Wallis test.

HBV = hepatitis B virus; HCV = hepatitis C virus; AST = aspartate aminotransferase; ALT = alanine aminotransferase; yGT = gamma-glutamyl transpeptidase; AP = alkaline phosphatase; INR = international normalized ratio; LDL = low density lipoprotein; HDL = high density lipoprotein; APRI = AST to platelet ratio index

Median liver stiffness measurement for 112 HIV/HCV co-infected patients was 6.9 kPa, while in the 24 HIV/HBV co-infected patients, it was 5.5 kPa (IQR: 4.5–10.3). The prevalence of alcohol overuse at the time of the study was found in 5.4% (18/333) of patients, while in the past it was found in 2.4% (8/333). Interestingly, alcohol overuse was reported in 12% of patients with significant fibrosis or cirrhosis. However, no significant differences were observed between the groups.

Interestingly, the prevalence for significant fibrosis or cirrhosis is higher among smokers, who make up the majority of the population with 38.1% (127/333) being the highest in HIV/HCV co-infected patients (Table 2). The levels of aminotransferases and other liver function tests (AST, ALT, γ-GT, AP, albumin INR, platelet count, cholesterol, HDL, LDL) reflected the expected liver injury and liver dysfunction (Table 2). Clinical manifestations of portal hypertension appeared in about 20% of the cohort as follows: 16.5% splenomegaly (55/333), 2.4% esophageal varices (8/333) and 1.3% ascites (5/333).

### Subgroup comparison of HIV mono-infected and HCV co-infected patients

S1 and S2 Tables depicts the main differences in clinical and demographic characteristics of HIV mono-infected compared to HIV/HCV co-infected patients. While gender, age and exposure to cART were similar in both groups (S1 Table), TE, APRI and FIB4-score were significantly higher in co-infected patients (S2 Table). Moreover, 22/202 (11%) of patients without hepatitis co-infection had a significant fibrosis and 4/202 (2%) had cirrhosis. Similarly, the laboratory parameters showed more severe liver damage and lower cholesterol levels in co-infected patients (S2 Table). As expected, the transmission route of HIV was different for the co-infected patients, with more transmission through transfusion and IVDA (S2 Table). In co-infected patients, there were more smokers and patients with alcohol overuse. Also, HCV co-infected patients showed longer cART-exposure and time from HIV diagnosis, but a lower CD4 cell-count and more CDC stage B (S2 Table).

While HCV-load was not significantly different across the fibrosis stage in the co-infected patients, nearly half of the patients in this cohort had been treated with interferon (IFN)-based therapies and 23% reached SVR (S3 Table). Co-infected patients with significant fibrosis or cirrhosis had been significantly more exposed to interferon therapies compared to the others (S3 Table). In HIV/HCV co-infected patients, genotype 1 was the most frequent one with 46% in all fibrosis groups. Remarkably, genotype 3 was significantly higher in cirrhotic patients (S3 Table). One patient each had a simultaneous co-infection with genotype 1 and 3 and genotype 1 and 4, respectively.

### Relationship of cART and liver fibrosis

The exposure to PI-based cART was associated with a higher rate of significant fibrosis, followed by NRTI-based and then NNRTI-based regimens (Fig 1C). Patients with significant fibrosis had experienced multiple therapy (more than two regimens) more frequently than those without cirrhosis or fibrosis (45% vs. 18.3%, p<0.001). About one third (33%; 58/333) of the cohort had actually or in the past been treated with AZT/ddI/D4T, while 32.4% (108/333) had been exposed to AZT, 25.8% (86/333) to D4T and 12.3% (41/333) to ddI. Of note, the prevalence of cirrhosis among patients with exposure to cART containing AZT was 68% compared to patients with significant fibrosis or without fibrosis (35% and 28.2%, p<0.001). Among the cirrhotic patients, a history of D4T (p = 0.009) and ddI (p<0.001) intake was significantly higher than in patients with significant or no fibrosis. Similarly, patients with a history of ddI and AZT showed higher levels of TE than patients without (Fig 1D and 1E).

More detailed analysis of HIV mono-infected patients revealed that 36% (72/202) had received AZT, ddI or D4T and 18% of those (13/72) had at least significant fibrosis or cirrhosis (S4 Table). In three patients, idiopathic non-cirrhotic portal hypertension was diagnosed due to variceal bleeding, while one patient had portal vein thrombosis without underlying disease. The only risk factor these three patients had in common was long-term exposure to ddI (mean exposure six years, range 6–7.5 yrs).

### Factors associated with significant fibrosis in mono- and co-infected patients

Uni-variate analysis identified HCV infection, history of AZT, history of ddI, more than two different cART regimes, nicotine abuse and hepatic steatosis to be significantly associated with significant fibrosis, while HIV-load < 40copies/ml seem to be protective (Table 3).

**Table 3:** Uni-variate analysis of factors associated with significant fibrosis (TE ≥ 7.1 kPa) according to logistic regression forward step-wise likelihood quotient in the whole cohort (n = 333).

Variable	Unadjusted OR (95% CI)	p-value
**HCV positive**	5.7 (3.4–9.7)	<0.001
**ddI**	3.3 (1.7–6.5)	<0.001
**AZT**	2.1 (1.2–3.4)	0.012
**more than two different cART-regimes**	1.9 (1.1–3.1)	0.004
**Smoking**	2.1 (1.2–3.6)	
**Fatty liver diagnosis in ultrasound**	1.8 (1.1–2.9)	0.04
**HIV<40c/ml**	0.5 (0.3–0.9)	<0.001

TE = transient elastography; kPa = kilopascal; OR = odds ratio; CI = confidence interval; HCV = hepatitis C virus; ddI = didanosine; AZT = azidothymidine; HIV = human immunodeficiency virus; HBV = hepatitis B virus; c/ml: copies/milliliter

Multivariate logistic regression analysis showed an independent association for HCV infection and history of ddI-intake with significant fibrosis, while HIV-load < 40copies/ml was associated with absence of significant fibrosis (Table 4).

**Table 4:** Multivariate analysis of the factors associated with significant fibrosis (TE ≥ 7.1 kPa) according to logistic regression forward step-wise likelihood quotient in the whole cohort (n = 333).

Variable	Adjusted OR (95% CI)	p-value
**HCV positive**	5.3 (3.1–9.1)	<0.001
**ddI**	2.7 (1.3–5.6)	0.008
**HIV<40c/ml**	0.5 (0.3–0.9)	0.024

TE = transient elastography; kPa = kilopascal; OR = odds ratio; CI = confidence interval; HCV = hepatitis C virus; ddI = didanosine; HIV = human immunodeficiency virus; c/ml: copies/milliliter

When analyzing as end-point the presence of cirrhosis assessed as TE ≥ 12.5kPa, HCV infection, history of ddI- and AZT intake were independently associated with cirrhosis (S5 Table). Since HCV-coinfection is an independent factor for the presence of cirrhosis, we then analyzed only HIV mono-infected patients, and in those history of ddI treatment had a very strong association with presence of cirrhosis. Next, when focusing on the in HCV co-infected patients, a history of AZT intake was independently associated with presence of cirrhosis (S5 Table).

## Discussion

This study demonstrates that history of ddI intake and AZT intake are independently associated with presence of significant fibrosis and cirrhosis. Importantly, sufficient suppression of HIV replication protected from significant liver fibrosis.

While life expectancy has increased in recent decades, liver disease in HIV-infected patients is an emerging concern in a population, where co-morbidities have become the main cause of death in recent years [1]. Advanced liver fibrosis is more frequent in HCV co-infected patients than in HIV- mono-infected patients [2, 22]. HIV/HCV co-infection is characterized by a more rapid progression to liver fibrosis compared to HCV mono-infection [23]. Importantly, as liver fibrosis progression may be blunted in HIV/HCV co-infected patients due to cART, there has been increasing concern regarding long-term use of antiretroviral therapy [11, 15, 17, 24]. In several studies, cART-induced hepatotoxicity and other risk factors, which might be causal for the development of liver fibrosis in HIV-infected patients, are controversially discussed [17]. While on the one hand, effective control of HIV was associated with slower liver fibrosis progression in HIV/HCV co-infected patients, especially through the use of PIs [3, 6, 25], exposure to ddI, on the other hand, has been associated with liver injury [5, 10, 16]. Moreover, nucleoside analog reverse-transcriptase inhibitors (NRTIs), e.g. stavudine (d4T), have also been blamed for liver injury [25, 26]. In our study, we show a very clear association of PI-based cART with incidence of significant liver fibrosis or cirrhosis. These effects occurred independent of other factors. Besides a direct hepatic effect of PIs themself, these findings might reflect the fact that PIs were traditionally started in late presenters. Importantly, with decreasing CD4 count, fibrosis progression is more likely. This has been shown previously [27] and also in our hands we observe a tendency, which is only significant if we compare HIV-mono-infected patients with HCV-co-infected patients. Therefore, the HIV drugs, which are preferably used in late presenters, may lead to the perception that they may be associated with more fibrosis progression, which however might not be necessarily causal. Importantly, ddI is especially associated with cirrhosis in mono-infected patients without other causes of liver injury.

Also, a high percentage (42%) of asymptomatic HIV mono-infected patients receiving cART might show abnormal TE values [28]. Further findings suggest that HIV directly interacts with hepatocytes, Kupffer cells and hepatic stellate cells, apart from indirectly affecting liver fibrosis through systemic immune activation with inflammation and reduced CD4 T-cell counts [29]. Although few data exist on the exact pathomechanism, HIV itself and/or long-term complications of cART possibly induces liver fibrosis in HIV mono-infected patients [17]. This line of evidence is also reflected in the increasing number of publications focusing on pathogenesis and clinical presentation of liver disease in HIV mono-infected patients [30, 31] and corresponds to our recently published findings, suggesting that HIV itself contributes to liver fibrosis and that in these patients, modern cART is protective rather than harmful [3]. This is confirmed by the present study, demonstrating that for the wide range of mono-infected patients, the strongest protection against development of liver fibrosis seems to be sufficient HIV-replication control, which outweighs the damage of previous cART regimens with drugs such as ddI and AZT.

The present study also shows that modern cART could be improving the liver phenotype of the patients, while even in co-infected patients, drugs such as d4T [5], previously assumed to induce liver fibrosis, are not independently associated with liver fibrosis. However, a strong association of ddI and AZT was still observed in cirrhotic patients in our study. Nevertheless, HIV/HCV co-infected patients face several additional risk factors, which might be associated with increased liver injury. Time since HIV-diagnosis is twice as long as in HIV mono-infected patients, stressing the possibility that HIV itself induced more injury in the liver. Moreover, immune reconstitution (CD4 count) seems to be less effective. Also, CDC stage is more advanced. Finally, these patients show more alcohol overuse and smoking. All together HIV-induced immunodeficiency drives the faster liver disease progression, which has been described to be more severe and progressive than in HCV mono-infected patients [32].

This study has several limitations. First, a cohort of healthy controls is missing to compare TE findings. However, TE is widely accepted and recently received FDA approval for this indication. Second, this is a cross-sectional study and repetitive TE measurements might uncover the dynamics of fibrosis. Third, didanosine might also cause non-cirrhotic portal hypertension, which might be missed by transient elastography. Further, although the cART-treatment was recorded, the time-point of CD4 nadir was not and the adherence to treatment could be demonstrated with precision. Moreover, some other important data, which might allow the calculation of fibrosis progression rate as published previously [27], are missing. Finally, the life style reporting was based on anamnestic questions and not a validated questionnaire, possibly resulting in underestimation of some features, e.g. alcohol overuse.

In summary, our study demonstrates that a history of ddI and AZT treatment still bears an unfavorable influence on the presence of liver disease, whereas control of HIV replication seems to be the master switch in hampering the development of liver fibrosis in HIV patients.

## Supporting information

S1 Table (DOCX)

S2 Table (DOCX)

S3 Table (DOCX)

S4 Table (DOCX)

S5 Table (DOCX)
