# Supplementary material for: Exposure to previous cART is associated with significant liver fibrosis and cirrhosis in human immunodeficiency virus-infected patients
Source: PLoS One. 2018 Jan 18;13(1):e0191118. doi: 10.1371/journal.pone.0191118 (PMC5773180; doi:10.1371/journal.pone.0191118)
Supplement: S1 Table — (DOCX) [file pone.0191118.s001.docx]

**Supplement table 1:** Clinical and demographic characteristics of HIV mono-infected and HIV/HCV co-infected patients.

|  | **HIV mono-infected**  **(n = 202)** | **HIV/HCV co-infected**  **(n = 112)** | **p-value** |
| --- | --- | --- | --- |
| **Male** | 162 (80%) | 97 (87%) | 0.172 |
| **Age [y]** | 46 (38-52) | 44 (39-50) | 0.372 |
| **Undetectable HIV load** | 163 (81%) | 85 (76%) | 0.316 |
| **Exposure to cART** | 177 (88%) | 101 (90%) | 0.581 |
| **Lipodystrophy** | 21 (10%) | 10 (9%) | 0.844 |
| **CD4 count < 200 cells/µl** | 22 (11%) | 7 (6%) | 0.223 |
| **Albumin [g/l]** | 43.4 (40.8-46.7) | 42.3 (38.4-44.7) | 0.126 |
| **BMI [kg/m^2^]** | 23 (21-26) | 23 (20-25) | 0.479 |
| **Hypertension** | 32 (16%) | 16 (14%) | 0.747 |

Data are shown as median and (interquartile range) or numbers and (%). Comparisons are performed using Mann-Whitney-U test.

BMI = Body-Mass-Index.
