# Supplementary material for: Exposure to previous cART is associated with significant liver fibrosis and cirrhosis in human immunodeficiency virus-infected patients
Source: PLoS One. 2018 Jan 18;13(1):e0191118. doi: 10.1371/journal.pone.0191118 (PMC5773180; doi:10.1371/journal.pone.0191118)
Supplement: S2 Table — (DOCX) [file pone.0191118.s002.docx]

**Supplementary table 2:** Clinical and demographic characteristics of HIV mono-infected and HIV/HCV co-infected patients

|  | **HIV mono-infected**  **(n = 202)** | **HIV/HCV co-infected**  **(n = 112)** | **p-value** |
| --- | --- | --- | --- |
| **TE [kPa]** | 5.2 (4.3-6.3) | 7 (5.3-10.3) | <0.001 |
| **APRI score** | 0.3 (0.2-0.4) | 0.6 (0.4-1.1) | <0.001 |
| **APRI score > 1** | 6 (3%) | 37 (33%) | <0.001 |
| **FIB4 score** | 0.9 (0.7-1.2) | 1.5 (0.9-2.5) | <0.001 |
| **FIB4 index > 1.45** | 35 (17%) | 58 (52%) | <0.001 |
| **AST [U/l]** | 23 (16-29) | 41 (30-62) | <0.001 |
| **ALT [U/l]** | 27 (20-35) | 49 (25-78) | <0.001 |
| **AP [U/l]** | 85 (69-109,8) | 92 (73-118) | 0.059 |
| **Bilirubin [mg/dl]** | 0.4 (0.3-0.7) | 0.6 (0.4-1.4) | <0.001 |
| **INR** | 1 (0.9-1) | 1 (1-1.1) | <0.001 |
| **Platelets [x10^3^G/l]** | 218 (179-254) | 179 (142-222) | <0.001 |
| **Total cholesterol [mg/dl]** | 195 (173-226) | 168 (140-192) | <0.001 |
| **LDL [mg/dl]** | 112 (80-136) | 91 (62-121) | 0.01 |
| **HDL [mg/dl]** | 42 (33-52) | 37 (31-48) | 0.099 |
| **Transmission of HIV** |  |  |  |
| **MSM** | 94 (47%) | 23 (21%) | <0.001 |
| **Heterosexual** | 59 (29%) | 5 (5%) | <0.001 |
| **Transfusion** | 9 (5%) | 32 (29%) | <0.001 |
| **IVDA** | 7 (4%) | 43 (38%) | <0.001 |
| **Unknown** | 33 (16%) | 9 (8%) | 0.039 |
| **Alcohol overuse** | 11 (5%) | 14 (13%) | 0.059 |
| **Smokers** | 110 (55%) | 82 (73%) | 0.001 |
| **Time since HIV diagnosis [y]** | 8 (4-13) | 16 (8-25) | <0.001 |
| **CDC stage (A / B / C)** | 106/ 46/ 49  (53/ 23/ 24%) | 52/ 38/ 21  (46/ 34/ 19%) | n.s./ 0.034/ n.s. |
| **Duration of cART [y]** | 4 (2-10) | 7 (2.5-13) | 0.057 |
| **CD4 count [cells/µl]** | 498 (356-649) | 423 (287-623) | 0.045 |

Data are shown as median and (interquartile range) or numbers and (%). Comparisons are performed using Mann-Whitney-U test.

MSM = men who have sex with men; IVDA = intravenous drug abuse; AST = aspartate aminotransferase; ALT = alanine aminotransferase; AP = alkaline phosphatase; INR = international normalized ratio; LDL = low density lipoprotein; HDL = high density lipoprotein; APRI = AST to platelet ratio index; CDC = Centers for Disease Control and Prevention; TE = transient elastography; kPa = kilopascal
