# Supplementary material for: Exposure to previous cART is associated with significant liver fibrosis and cirrhosis in human immunodeficiency virus-infected patients
Source: PLoS One. 2018 Jan 18;13(1):e0191118. doi: 10.1371/journal.pone.0191118 (PMC5773180; doi:10.1371/journal.pone.0191118)
Supplement: S3 Table — (DOCX) [file pone.0191118.s003.docx]

**Supplement table 3:** Main characteristics of HIV/HCV co-infected patients (n = 112).

| **Variable** | **All patients**  **(n = 112)** | **TE < 7.1 kPa**  **(n = 58)** | **7.1≤TE<12.5kPa**  **(n = 35)** | **TE≥12.5kPa**  **(n = 19)** | **p-value** |
| --- | --- | --- | --- | --- | --- |
| **HCV-RNA [10^6^ Copies/ml]** | 1.0 (0.003-3.8) | 1.1 (0.001-3.9) | 0.8 (0.001-3.1) | 0.8 (0.0001-2.5) | 0.911 |
| **Prior/current IFN-based therapy** | 52 (46 %) | 19 (33 %) | 22 (63 %) | 11 (58 %) | 0.007 |
| **SVR achieved** | 26 (23%) | 14 (24%) | 8 (24%) | 4 (21%) | 0.970 |
| **Transient elastography [kPa]** | 6.9 (5.3- 10.6) | 5.3 (4.5- 6.1) | 9.0 (8.4- 10.7) | 21.3 (15.1- 39.6) | 0.001 |
|  |  |  |  |  |  |
| **HCV-genotype, n,(%)** |  |  |  |  |  |
| **1** | 52 (46%) | 27 (47%) | 17 (49%) | 8 (42%) | 0.823 |
| **2** | 5 (5%) | 2 (3%) | 1 (3%) | 2 (11%) | 0.292 |
| **3** | 14 (13%) | 5 (9%) | 3 (9%) | 6 (32%) | 0.026 |
| **4** | 9 (8%) | 4 (7%) | 4 (11%) | 1 (5%) | 0.951 |
| **Unknown** | 34 (30%) | 21 (36%) | 11 (31%) | 2 (11%) | 0.052 |

Data are shown as median and (interquartile range) or number and (%). Comparisons are performed using Kuskal-Wallis test.

TE=Transient elastography; kPa = Kilopascal.
