# Supplementary material for: Exposure to previous cART is associated with significant liver fibrosis and cirrhosis in human immunodeficiency virus-infected patients
Source: PLoS One. 2018 Jan 18;13(1):e0191118. doi: 10.1371/journal.pone.0191118 (PMC5773180; doi:10.1371/journal.pone.0191118)
Supplement: S4 Table — (DOCX) [file pone.0191118.s004.docx]

**Supplement table 4:** Liver and HIV characteristics of HIV mono-infected patients with different cART and without alcohol overuse.

|  | **History of AZT-, DDI- or D4T**  **N = 66** | **No history of AZT-, DDI- or D4T**  **N = 125** |
| --- | --- | --- |
| **TE-value [kPa]** | 5.3 (4.6-6.7) | 5.0 (4.3-6.1) * |
| **TE ≥ 7.1 kPa** | 9 (13.6%) | 11 (9%) * |
| **TE ≥ 12.5 kPa** | 2 (3%) | 1 (0.8%) * |
| **APRI-Score** | 0.3 (0.2-0.4) | 0.3 (0.2-0.4) |
| **APRI-Score > 1** | 3 (5%) | 3 (3%) |
| **FIB4-Score** | 1 (0.7-1.3) | 0.9 (0.7-1.2) |
| **FIB4-Score > 1.45** | 13 (21.7%) | 19 (17%) |
| **Liver injury [> 2x ULN AST]** | 1 (1.5%) | 1 (0.8%) |
| **Median HIV-load [Copies/ml]** | < 40 | 6,200±22,530.3 |
| **HIV-load < 40 copies/ml** | 60 (90%) | 94 (75%) * |
| **Median CD4-count [cells/µl]** | 553±298 | 492±218 |
| **CD4-count < 200/ µl** | 10 (15%) | 10 (8%) |

Data are shown as median and (interquartile range) or numbers and (%). Comparisons are performed using Mann-Whitney-U test.

AZT = Azidothymidine; DDI = Didanosine; D4T = Stavudine; cART = combined Antiretroviral Therapy; TE = Transient Elastography; kPa = Kilopascal; IQR = nterquartile Range; Apri = AST to Platelet Ratio Index; Fib4 = Fibrosis 4; ULN = Upper limit norm; AST = Aspartat Amino-Transferase; HIV = Human Immunodeficiency Virus.
