# Supplementary material for: Exposure to previous cART is associated with significant liver fibrosis and cirrhosis in human immunodeficiency virus-infected patients
Source: PLoS One. 2018 Jan 18;13(1):e0191118. doi: 10.1371/journal.pone.0191118 (PMC5773180; doi:10.1371/journal.pone.0191118)
Supplement: S5 Table — (DOCX) [file pone.0191118.s005.docx]

**Supplementary table 5:** Multivariate analysis of the factors associated with cirrhosis (TE ≥ 12.5 kPa) according to logistic regression forward step-wise likelihood quotient in the whole cohort (n=333), HIV-monoinfected (n=202) and HCV-coinfected-patients (n=112).

|  | **All patients** | | **HIV mono-infected** | | **HCV co-infected** | |
| --- | --- | --- | --- | --- | --- | --- |
| **Variable** | **OR (95% CI)** | **p** | **OR (95% CI)** | **p** | **OR (95% CI)** | **p** |
| **HCV positive** | 5.8 (2.2-15.4) | <0.001 |  |  |  |  |
| **ddI** | 2.9 (1.1-8.0) | 0.049 | 20.8 (1.2-37) | 0.038 |  |  |
| **AZT** | 2.8 (1.0-8.0) | 0.038 |  |  | 4.1 (1.2-13) | 0.021 |

TE = transient elastography; kPa = kilopascal; OR = odds ratio; CI = confidence interval; HCV = hepatitis C virus; ddI = didanosine; AZT = azidothymidine; HIV = human immunodeficiency virus; HBV = hepatitis B virus; c/ml: copies/milliliter
